# Food Insecurity, Walkability, and Social Determinants of Health: A Cross-Sectional, County-Level Study of Associations with Maternal and Infant Mortality in the United States

**DOI:** 10.3390/healthcare13192407

**Published:** 2025-09-24

**Authors:** Brooklyn Stone, Azita Amiri, Shuang Zhao, Shima Hamidi, Paige Johnson, Debra Bruns

**Affiliations:** 1 College of Nursing, The University of Alabama in Huntsville, Huntsville, AL 35805, USA; 2 Capstone College of Nursing, The University of Alabama, Tuscaloosa, AL 35487, USA; 3 Department of Political Science, The University of Alabama in Huntsville, Huntsville, AL 35899, USA; 4 Bloomberg School of Public Health, Johns Hopkins University, Baltimore, MD 21205, USA

**Keywords:** maternal mortality, infant mortality, social determinants of health, SDOH, food insecurity, walkability, education, food assistance, distance to obstetrics, mental distress, race, marital status, household income

## Abstract

Background: Compared to other high-income countries, US women face higher maternal and infant mortality rates. Methods: This study used structural equation modeling (SEM) to examine cross-sectional, county-level associations between structural and intermediary social determinants of health (SDOHs) and maternal and infant mortalities, based on the World Health Organization’s Commission on Social Determinants of Health framework. Results: Our findings suggest maternal mortality may be linked to increased food insecurity, food assistance, distance to obstetric care, and decreased walkability and marriage rates. Our modeling also points toward a connection between infant mortality and increased food insecurity, food assistance, Black race, mental distress, and decreased walkability, education, and income. SEM revealed significant direct and indirect effects of these SDOHs. Notably, food insecurity and walkability had direct associations with both maternal and infant mortality in both SEM models. Conclusions: The findings underscore the need for policy, practice, and research efforts to address key SDOHs and reduce mortality disparities in the US.

## 1. Introduction

Maternal and infant mortalities continue to be a significant problem in the United States (US). The World Health Organization defines maternal death as the annual number of female deaths from any cause related to or aggravated by pregnancy or its management (excluding accidental or incidental causes) during pregnancy and childbirth or within 42 days of termination of pregnancy, irrespective of the duration and site of the pregnancy, expressed per 100,000 live births, for a specified time period [1]. Maternal mortality rates decreased from 32.9 deaths per 100,000 live births in 2021 to 22.3 in 2022 [2], and early estimates for 2023 are revealing approximately 19 provisionally [3]. Even with the recent decline to pre-COVID-19 rates (2018–2019), the US continues to lead in maternal deaths for high-income nations [4]. Black women had the highest maternal mortality rates in the US in 2022. at 49.5 deaths per 100,000 live births, followed by White women with 19, Hispanic women with 16.9, and Asian women with 13.2 [2].

US women aged 40 and older had the highest maternal mortality among age groups in 2022, at 87.1, and the rate for women younger than 25 was 14.4 in 100,000 live births [2]. The Maternal Mortality Review Committee (MMRC) revealed that 53% of pregnancy-related deaths happened in the postpartum period from 7 days to 12 months following birth, using maternal mortality data from 36 US states from 2017 to 2019 [5]. In the same report, the MMRC also found that 84.2% of those maternal deaths were preventable [5]. The most frequent cause of pregnancy-related death was attributed to mental health conditions, at 22.7%, followed by hemorrhage (13.7%), cardiovascular conditions (12.8%), infection (9.2%), thrombotic embolism (8.7%), and cardiomyopathy (8.5%) [5]. The US states with the highest maternal mortality rates for 2018 to 2022 were Tennessee (41.1), Mississippi (39.1), Alabama (38.6), Arkansas (38.3), and Louisiana (37.3) [6].

The infant mortality rate in the US in 2022 was 5.61 infant deaths per 1000 live births, which increased by 3% from 5.44 in 2021 [7]. Infant mortality is the death of an infant before their first birthday per 1000 live births [8]. Congenital disabilities (19.5%), preterm birth or low birth weight (14%), sudden infant death syndrome (7.4%), injuries (6.6%), and maternal pregnancy complications (5.9%) were the leading causes of infant death in the US in 2022 [7]. The highest infant mortality rates in 2022 were noted among Black women, at 10.90 [7]. The US states with the highest infant mortality rates in 2022 were Mississippi (9.11 per 1000 live births), South Dakota (7.77), Arkansas (7.67), Delaware (7.49), and Louisiana (7.37). Adolescent mothers have been shown to have a higher risk of infant mortality compared to adult mothers [9].

The social determinants of health (SDOHs) are critical in improving health; socioeconomic status and the conditions in which they live their lives are so important they determine half of all health outcomes [10,11]. Mothers with symptoms of depression are more likely to experience infant mortality compared to mothers without depression [12]. The Conceptual Framework for Action on the Social Determinants of Health developed by the World Health Organization (WHO) and the Commission on Social Determinants of Health (CSDH) was initially used to guide the study by assisting with determining essential structural and intermediary variables for the mediation models [13]. Structural determinants of health are socioeconomic factors such as income, education, occupation, social class, gender, and race in the framework [13]. Intermediary determinants are material, psychosocial, behavioral, biological, and health system factors. Further information is needed to identify complex associations between structural and intermediary determinants of health and maternal mortality and infant mortality in the US.

Hagatulah et al. [14] found an association between postpartum depression (PPD) and an increased risk of death of both natural and unnatural causes compared to women who did not have PPD. Postpartum women with severe psychiatric disorders had an increased risk of maternal mortality [15,16]. The risk for suicidality (deaths, attempts, and ideation) is increased for pregnant and postpartum women with depression, and suicide is a leading contributor to maternal mortality among depressed women during this time [14,17,18].

Obstetrics units in hospitals in rural areas in the US have experienced closures, and as a result, births outside of a hospital or in a hospital without obstetrics and preterm births have increased for women living in rural areas that have experienced these losses of obstetrics services [19]. According to Hung et al. [20], 9% of counties in the US completely lost their obstetrics services between 2004 and 2014, and another 45% of rural counties had no obstetrics services during this time. Pregnant women living in rural areas are at increased risk of being admitted to the ICU and maternal mortality than women residing in urban areas [21]. India-Aldana et al. [22] found that women who lived in more walkable neighborhoods had lower risk of obesity-related mortality. The risk for preterm birth, low birth weight, gestational diabetes, and hypertension are decreased for pregnant women living in areas with higher walkability [23]. Non-gestational or pre-gestational diabetes increases the risk of infant mortality [24]. Adults who had very low food insecurity had two times the risk of all-cause mortality compared to food-secure adults [25]. Food insecurity and diabetes were associated with increased infant mortality [26]. There is a lack of studies conducted in the US examining the associations between food insecurity and maternal and infant mortality as well as walkability and maternal and infant mortality. Literature on maternal and infant mortalities must consider the interrelated associations between structural and intermediary determinants of health affecting maternal and infant mortalities in the US, but it is currently missing. The aim of this study is to examine the associations between structural and intermediary SDOHs and maternal and infant mortality rates across US counties. Specifically, the study seeks to understand how variables such as income, education, race, food insecurity, walkability, mental distress, and distance to obstetric services are directly and indirectly associated with maternal and infant mortality, using structural equation modeling (SEM) to capture complex, interrelated effects.

**Figure 1:**
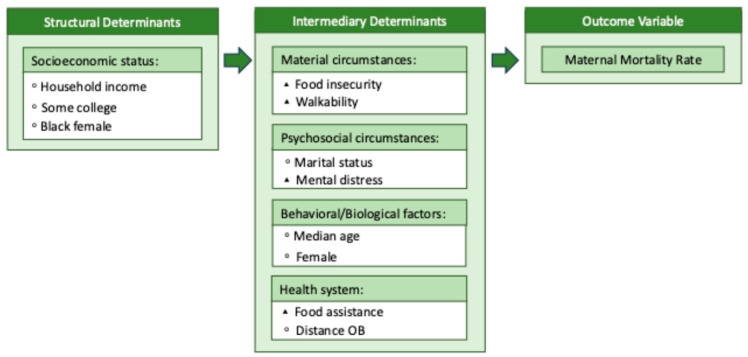
Model 1 conceptual framework. Note: This figure is a modified version of the CSDH conceptual framework. The conceptual framework will be used as a guide for the secondary data analysis of Model 1. ◦ = Exogenous variable, ▴ = endogenous variable.

**Figure 2:**
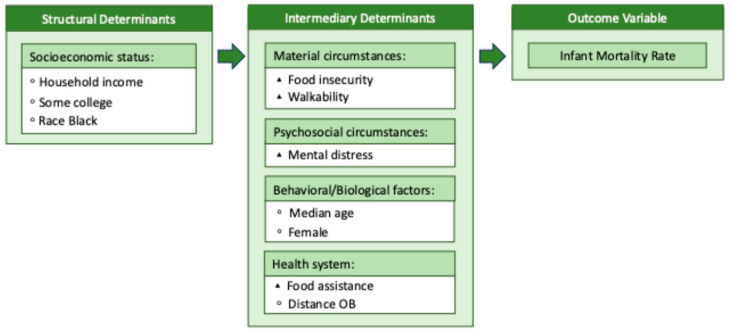
Model 2 conceptual framework. Note: This figure is a modified version of the CSDH conceptual framework. The conceptual framework will be used as a guide for the secondary data analysis of Model 2. ◦ = Exogenous variable, ▴ = endogenous variable.

Modified versions of the CSDH conceptual framework are presented in Figure 1 and Figure 2 with all respective variables of interest.

## 2. Materials and Methods

A cross-sectional secondary data analysis investigated the associations between structural and intermediary determinants of health and maternal and infant mortality rates. This study analyzes county-level maternal and infant mortality rates across the United States, using data from national sources including the CDC WONDER, National Center for Health Statistics (NCHS), and County Health Rankings, with the population defined as all live births occurring between 2015 and 2020 in counties meeting reporting thresholds (≥10 deaths), as determined by CDC suppression guidelines. All of the variables included in Model 1 and Model 2 and the sources for the data are presented in Table 1 and Table 2, respectively. The data in this analysis is being analyzed at the county level in the US for all 50 states. All variables will be referred to as exogenous or endogenous in Model 1 and Model 2.

### 2.1. Variables

#### 2.1.1. Maternal Mortality Rate

The maternal mortality rate in this study came from the Agency for Healthcare Research and Quality (AHRQ) database, sourced from the CDC and Prevention Wide-ranging Online Data for Epidemiologic Research (WONDER) and National Center for Health Statistics (NCHS) [27]. This variable is the total number of maternal deaths per 100,000 population (100,000 live births). The CDC suppresses data for counties with less than ten deaths, which may potentially cause underestimation of mortality.

#### 2.1.2. Infant Mortality Rate

The infant mortality rate in this study is defined by the number of infant deaths (within 1 year) per 1000 live births. This variable was downloaded from County Health Rankings (CHR) data, and the source was the NCHS Natality and Mortality Files and the National Vital Statistics System (NVSS) [28]. It is important to note that infant mortality is a rare event in terms of statistical analysis, and the CDC suppresses any rates when there are fewer than ten infant deaths in a county. As a result, there can be an underestimation of mortalities.

#### 2.1.3. Structural Determinants

Annual median household income is where half of a county’s households earn more and half of households earn less. CHR and ACS are the sources for this variable [28]. Some college is defined as the percentage of the population ages 25–44 with some post-secondary education, such as enrollment in a vocational/technical school, junior college, or 4-year college. This variable comes from CHR and the ACS [28]. Race Black is the percentage of the population who report Black or African American race only. This variable comes from the AHRQ SDOH database and the ACS [27]. Black female is the percentage of the Black or African American population who report to be female. The AHRQ SDOH database and the ACS are the sources for this variable [27]. It is important to note that race in this study is a structural determinant of health and, therefore, is a measure of social variation and is not based on biological differences.

#### 2.1.4. Intermediary Determinants

This study defines walkability by the National Walkability Index (NWI) score. The US Environmental Protection Agency (EPA) is the data source [29]. The NWI uses measures of intersection density, distance to transit stops, and diversity of land uses (employment mix and employment and household mix) to determine scores by ranking these measures. The scoring of the NWI is 1–5.75, which is the least walkable; 5.76–10.5, which is below average walkable; 10.51–15.25, which is above average walkable; and 15.26–20, which is most walkable. This variable is available at the block group level; therefore, the means of the tracks were calculated to obtain the average walkability for each county. Because walkability is considered a relatively stable, built-environment measure that does not change substantially year to year, the 2019 index was used as a proxy across the 2015–2020 study period.

The median distance to obstetrics is defined as the median distance in miles to the nearest obstetrics department in a hospital. This variable was calculated using population-weighted tract centroids in the county. The variable was downloaded from the AHRQ SDOH database and was initially sourced from Provider of Services Files collected in the Centers for Medicare & Medicaid Services provider certification process [27].

Food insecurity is expressed as the percentage of the population who did not have access to a reliable source of food in the past 12 months. This variable was sourced from CHR and Feeding America Map the Meal Gap [28]. Food assistance is the percentage of households that received food stamps/SNAP in the past 12 months. This variable came from the AHRQ SDOH database and the ACS [27].

Mental distress in this study is defined as the percentage of adults who reported 14 or more days in response to the following question: Now, thinking about your mental health, which includes stress, depression, and problems with emotions, for how many days during the past 30 days was your mental health not good? This variable is from the CHR and Behavioral Risk Factor Surveillance System (BRFSS), which surveys the total non-institutionalized population 18 years and older, asking questions about health behaviors and quality of life [28].

### 2.2. Statistical Analyses

StataNow/SE 18.5 was used for all statistical analyses in this study. Descriptive statistics are shown in Table 3, including frequencies, means, and standard deviations for all variables in Model 1 and Model 2. Correlation statistics of all variables in each model were conducted, and simple bivariate analyses were performed to assess collinearity among the variables. All variables were transformed by natural log (In) to account for variances and improve estimation.

SEM, also known as covariance structure modeling, is used to assess the linear (direct) and non-linear (indirect) associations between the intermediary determinants, structural determinants, and outcome variables [30]. The variance in the outcome variables of maternal and infant mortality rates is explained by the structural and intermediary determinant variables using SEM [30]. SEM is achieved through a series of equations measuring the relationship between each variable, which is indicated by a solid straight arrow. Exogenous variables are given values that may influence endogenous and outcome variables in the model; however, exogenous variables are not influenced by other variables in the SEM [31]. Endogenous variables, or mediating variables, may be influenced by other variables in the SEM, mediating the association between the exogenous variables and the outcome variables [31].

**Figure 3:**
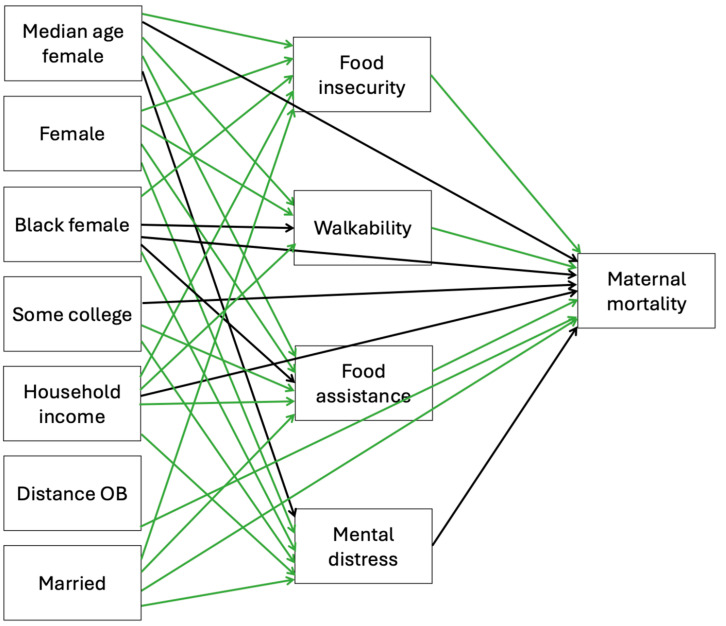
Associations between exogenous and endogenous social determinants of health variables and maternal mortality using SEM Model 1. Green arrow = significant, black arrow = not significant.

**Figure 4:**
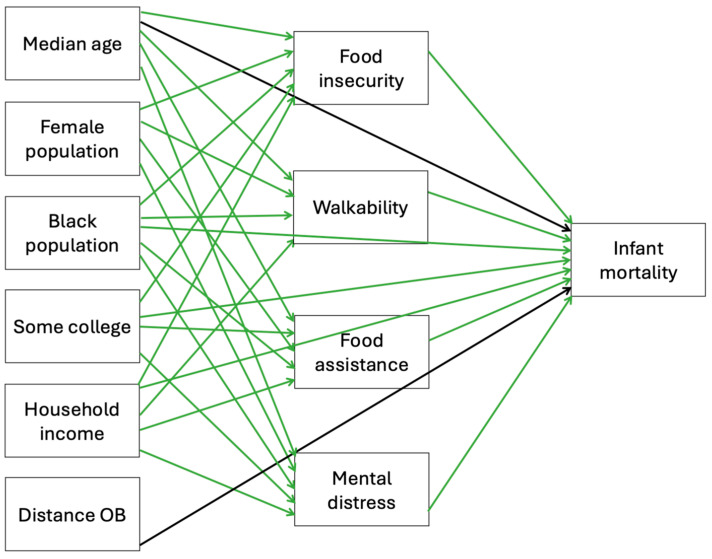
Associations between exogenous and endogenous social determinants of health variables and infant mortality using SEM Model 2. Green arrow = significant, black arrow = not significant.

A mediation model was created for Model 1 (SDOHs and maternal mortality rate) and Model 2 (SDOHs and infant mortality rate) based on previous literature surrounding the variables of interest in each model. The mediation models, complete with exogenous, endogenous, and outcome variables (maternal or infant mortality rates), were tested using SEM through Stata. The exogenous variables in Models 1 and 2, shown in Figure 3 and Figure 4, were chosen because they are outside the system and are given variables, influencing the endogenous and outcome variables. The exogenous variables are not influenced by other variables in the SEM. Endogenous variables in Models 1 and 2 are influenced by exogenous variables and act as mediators between the exogenous and outcome variables.

Exogenous variables in Model 1 are median age female, female, Black female, some college, household income, distance OB, and married. Median age, female population, Black population, some college, household income, and distance OB are the exogenous variables in Model 2. The exogenous variables are the boxes in the far-left column in Models 1 and 2. Endogenous variables are the boxes in the middle column of the models, and the outcome variables are the single boxes located in the far-right column in Models 1 and 2. The placement of all of the variables in the models is intentional and affects the associations analyzed in the models. These factors shape mental health within the system being analyzed, meaning mental health is not fixed or externally imposed (exogenous) but rather is explained by the model (endogenous). Mediating pathways in Model 1 connecting the endogenous variables with maternal mortality were food insecurity, food assistance, walkability, and mental distress. For example, mental distress is generally considered an endogenous variable, and it was treated as an endogenous variable in the study because it is influenced by social determinants (e.g., income, education, food insecurity) while also serving as a mediator between these determinants and the outcomes of maternal and infant mortality [32,33]. Mediating pathways in Model 2 connecting the endogenous variables with infant mortality were food insecurity, food assistance, walkability, and mental distress. Fit statistics are reported to evaluate model performance. Statistical significance was defined as a *p*-value < 0.05.

## 3. Results

Descriptive statistics for the variables in Model 1 and Model 2 are presented in Table 3. The average median age was 41.2 for the total population, and among females it was 42.6. Females accounted for an average of 49.9% of the total population by county, and the Black race accounted for an average of 9%. The average of Black females among the Black population was 41.86%. The average number of married females was 48.7%. The average annual median household income was $50,122 among the total population. An average of 14.12% of the population had food insecurity and lacked a reliable food source in the previous 12 months. Walkability averaged an NWI score of 6.5, which is below average. The average median distance to a hospital with obstetrics services was 11 miles. An average of 12.6% of adults reported having had mental distress (stress, depression, and problems with emotions) 14 or more days over the previous 30 days. An average of 57% of the population ages 25–44 had some post-secondary education (enrollment in a vocational/technical school, junior college, or 4-year college).

The models with the best fit are presented in Figure 3 and Figure 4. R-squared and Bentler–Raykov squared multiple correlation coefficients were used to measure fit in Stata. In Model 1, the R-squared value was 0.96, meaning the model predicted 96% of the associations in the model, and the Bentler–Raykov squared multiple correlational coefficient value was 0.83, meaning the model explained 83% of the variance in the outcome variable of maternal mortality. In Model 2, the R-squared value was 0.83, meaning the model predicted 83% of the associations in the model, and Bentler–Raykov squared multiple correlational coefficient value was 0.39, meaning the model explained 39% of the variance in the outcome variable infant mortality.

Direct effects of the variables in the models and regression coefficients are provided in Table 4 and Table 5. In Model 1, 23 of the 31 direct observed relationships between the variables in the model are highly significant. Median age female is directly and significantly associated with food insecurity, food assistance, and walkability. A lower median age among the female population may be associated with increased food insecurity and walkability. A higher median age among females has an observed association with increased food assistance. More females in the population may be associated with increased food assistance, walkability, and increased mental distress. Fewer females may be associated with more food insecurity. Less college education may be associated with increased food assistance and mental distress.

Lower household income may be associated with more food insecurity, food assistance, and mental distress, while higher household income may be associated with more walkability. Increases in females in the Black population are associated with increases in food insecurity and mental distress. The model suggests individuals who live farther away from obstetrics services have increased maternal mortality rates. Unmarried women in the model have observed associations with increased food insecurity, food assistance, mental distress, and maternal mortality. Mediating variables food insecurity, food assistance, and walkability have a significant observed association with maternal mortality rate. Greater food insecurity and food assistance may be associated with increased maternal mortality rates, and less walkability may be associated with increased maternal mortality rates.

Model 2 has 26 highly significant observed associations with the 28 equations in the model. A lower median age may be associated with more food insecurity, food assistance, mental distress, and walkability. An increase in females appears to be associated with increased food insecurity, food assistance, mental distress, and walkability. Fewer individuals of the Black race has an observed association with less walkability, and more individuals of the Black race has an observed association with more food insecurity, food assistance, mental distress, and infant mortality rates. Less college education may be associated with increased food insecurity, food assistance, mental distress, and infant mortality.

Lower household income has an observed association with higher food insecurity, food assistance, mental distress, and infant mortality; in contrast, higher household income has an observed association with greater walkability. The endogenous variables are food insecurity, food assistance, walkability, and mental distress. The model suggests increased food insecurity, food assistance, and mental distress may be associated with increased infant mortality rates. Less walkability has an observed association with increased infant mortality rates.

The direct, indirect, and total effects of the exogenous and endogenous variables on maternal mortality (Model 1) and infant mortality (Model 2) are presented in Table 6 and Table 7. Median age female and Black female have significant positive indirect effects on maternal mortality. Female, marital status, and household income have substantial negative indirect impacts on maternal mortality. Some college and household income are both significantly and negatively associated with infant mortality, while Black race is significant but positively associated with infant mortality.

## 4. Discussion

SDOHs have been an increasing topic of interest in recent years due to the enormous impact these determinants have on our health, from socioeconomic factors such as race, gender, income, and education level to the environment that surrounds us daily and over time [10,11]. This study is novel in the approach of using SEM to examine the complex associations between structural and intermediary SDOHs and their impact on maternal mortality and infant mortality through direct, indirect, and total effects that explain the intricate network of these SDOHs. The findings from Model 1 revealed that increased food insecurity, increased percentage of people who receive food assistance in the past year, decreased walkability, increased distance in miles to the nearest hospital with obstetrics services, and reduced percentage of married women are significantly associated with increased maternal mortality rates. Model 2 showed higher infant mortality rates are associated with increases in food insecurity in the previous 12 months, the percentage of people receiving food assistance, the percentage of the Black female population, and the percentage of adults reporting frequent mental distress, as well as decreases in post-secondary education, annual median household income, and walkability.

Model 1 demonstrated strong predictive capacity, with 96% of the hypothesized associations supported by the data. The model accounted for 83% of the variance in maternal mortality, indicating that area-level factors such as food insecurity, walkability, and access to obstetric care explain a substantial proportion of differences in maternal mortality across US counties. However, approximately 17% of the variance remains unexplained, suggesting that additional unmeasured factors continue to contribute to maternal mortality, highlighting the influence of additional factors such as individual health risks, systemic inequities, and quality of care that were not captured in this analysis. In Model 2, the model predicted 83% of the associations, and the model explained 39% of the variance in the outcome variable of infant mortality, though a majority of the variance remains unexplained and may reflect additional social, environmental, and healthcare-related influences. Many of the findings in this study are supported by prior literature. We found that food insecurity was essential to maternal and infant mortality and directly related. Evidence supports that increased food insecurity is associated with increased infant mortality [26]. Food insecurity has been shown to increase the risk of all-cause and cardiovascular disease mortality in adults [25,34]; however, there is a gap in the literature to support an association between food insecurity and maternal mortality specifically. Therefore, these findings are valuable.

Women living in more walkable neighborhoods have a lower risk of obesity-related mortality [22]. In Model 1, we expand existing knowledge to add that less walkability was significantly associated with increased maternal mortality. Less walkability was also significantly associated with increased infant mortality rates in Model 2. Conway and Menclova [23] found that pregnant women living in walkable neighborhoods had better health and birth outcomes. The connection between less walkability and increased infant mortality seems to be a new and insightful finding from this analysis. It is important to acknowledge that walkability may be acting as a proxy for unmeasured individual-level health behaviors such as obesity, diet, or physical activity. Therefore, the association observed here should not be interpreted as evidence of a direct causal pathway between the built environment and mortality. Instead, our findings suggest that walkability and, by extension, the built environment, can serve as an area-based marker of broader structural and lifestyle-related risk factors that contribute to maternal and infant health inequities. Future studies that integrate both individual-level health behavior data and contextual built environment measures are warranted to clarify these pathways.

Fewer married women is significantly associated with increased maternal mortality rates based on its substantial impact on the mediating variable of food insecurity in our study. Hanson et al. [35] found that unmarried individuals had higher food insecurity. A report from the NCHS found that food insecurity was increased in unmarried people living with children [36]. Unmarried women may lack the financial resources to acquire food for themselves and their families, as food insecurity was also associated with infant mortality in Model 2. This economic hardship may be due to one income, as more married women was associated with higher household income in Model 1. Unmarried women was directly associated with increased food assistance and indirectly associated with increased maternal mortality through the mediating variable of food assistance.

Food assistance was directly associated with increased maternal and infant mortalities in Models 1 and 2. Lower household income, less college education, fewer married women, and more older women had direct and robust impacts on increased food assistance and were indirectly associated with increased maternal mortality through food assistance. Studies have shown that food assistance through food stamps programs (SNAP and WIC) increases positive health outcomes [37] and lower mortality [38]; however, Conrad et al. [39] found that families receiving SNAP had a higher risk of mortality (all-cause, cardiovascular, and diabetes). We speculate that diet may play a role in the association between increased food assistance and maternal and infant mortalities. Leung et al. [40] found that increased food insecurity and SNAP participation were associated with a diet high in unhealthy processed foods. Other studies also support the association between an unhealthy diet with more processed sugar products and fewer fruits and vegetables among SNAP participants [41,42,43,44].

In Model 1, fewer females was associated with increased food insecurity, while Model 2 found more females was associated with increased food insecurity, and in turn, increased food insecurity was associated with increased maternal mortality and infant mortality. The variation among females and food insecurity represents how the inclusion of different SDOHs and outcome variables interact in each model. It is essential to point out that many of these structural and intermediary SDOHs are interrelated to one another, thus making it harder to accurately isolate the associations between them. To further describe the interrelation between the SDOH variables in this study, household income, for example, was directly and significantly associated with infant mortality and not directly and significantly associated with maternal mortality; however, household income did have indirect and significant associations with infant and maternal mortalities through food insecurity, food assistance, walkability, and mental distress. By modeling mental distress as an endogenous variable, we acknowledge its dual role as both a consequence of social disadvantage and a determinant of adverse maternal and infant outcomes. This specification aligns with social determinant frameworks [13] and provides a more comprehensive understanding of the mechanisms leading to maternal and infant mortality [32,33].

Food insecurity and walkability are key intermediary variables in this study. Both are shaped by broader structural factors such as income, education, and neighborhood resources. In turn, they may mediate the relationship between these structural determinants and maternal and infant mortality outcomes. By explicitly considering their dual role as outcomes of structural factors and as potential mediators, SEM allows for a more nuanced understanding of the pathways linking social determinants to health outcomes. Food insecurity can be due to financial and physical limitations; therefore, employment, household income, marital status, education, disability, walkability, and distance to grocery stores are all other SDOHs that may impact food insecurity, and each of these SDOHs impacts other SDOHs, which in turn determine 50% of our health outcomes [10,11]. In addition, people living in less walkable neighborhoods may not have the physical ability to access food, leading to food insecurity, which in turn can impact mortalities in the case of pregnant or postpartum women and their children. Chung et al. [45] found that people living in walkable communities had a lower risk of food insecurity due to physical limitations. We theorize that living in a less walkable neighborhood may impact food insecurity and the need for food assistance due also in part to financial hardship, as living in more walkable neighborhoods was associated with increased household income in Model 1. Conderino et al. [46] found variance in the association between walkability and income, revealing that White and Black people of low income had lower walkability. This study shows that exogenous social determinants may have direct and indirect impacts working through the endogenous social determinants to effect maternal and infant mortalities. This network of complicated relationships makes SEM the appropriate statistical analysis to use.

### Implications for Policies, Practice, and Future Research

In this cross-sectional secondary data analysis examining structural and intermediary social determinants of health and maternal and infant mortalities using county-level data from 2015 to 2020, we found that several of the crucial structural and intermediary determinants of health included in this analysis were significantly associated with maternal and infant mortalities. Food insecurity was a significant focus, and the findings of this study have a substantial impact on maternal and infant mortality. Healthy People 2030 [47] suggests that increasing enrollment and the amount provided in food assistance programs, as well as improving unemployment rates, would help decrease food insecurity. When considering improving food assistance programs, addressing some of the current problems related to unhealthy diets among SNAP participants is essential. The American Medical Association (AMA) recommends expanding enrollment in the Farmer’s Market Nutritional Program (FMNP) and the Women, Infants, and Children (WIC) program that encourage the consumption of foods with nutrients instead of processed foods with little nutritional value. Another strategy is closing the price gap between unhealthy, more affordable and healthy, more expensive foods. Revising the current policies within SNAP to increase the value given to accommodate the increased amount of more nutritious foods while also stipulating what can be purchased and excluding processed food with less nutritional value should be considered.

Unemployment and education, including skills training, should be prioritized when trying to improve household income and thus food insecurity, maternal mortality, and infant mortality. Walkability is another vital central variable in this study. Improving and increasing the number of sidewalks, increasing pedestrian safety through efficient traffic lights and signage [48], and narrowing lanes to accommodate sidewalks and bike lanes in addition to safety [49] are strategies that local communities and their leaders should address for improved walkability. Increasing walkability may help make it possible for people to increase their activity level [50] while increasing access to the community [51], reducing vehicle traffic [51], and increasing opportunities for social interaction [50]. Federal and local governments should incorporate these structural and intermediary SDOHs into their plans when strategizing policy changes to improve maternal and infant mortality.

Healthcare providers’ increased awareness of the impact that food insecurity and the other SDOHs can have on women and children is important when assessing patients, as several of the SDOHs in this analysis have been shown to impact maternal and infant mortality rates. However, these structural and intermediary SDOHs are also integral when considering overall health and quality of life. Therefore, screening for these SDOHs should be prioritized. There are several screening tools available for SDOHs, such as the Social Needs Screening Tool from the American Academy of Family Physicians Foundation [52] and the Accountable Health Communities Health-Related Social Needs Screening Tool from the Centers for Medicare & Medicaid Services [53]. Once structural and intermediary SDOH risks have been identified, the next step is to provide patients with resources both health-related and outside of the health system as needed [54]. For example, a patient who reports food insecurity should be connected with resources such as information on food assistance programs, local food pantries, or community unemployment agencies if they need work.

While we used county-level data for this study, census tract level would have been ideal for this national study, as it would have provided more granular data than county-level data. Variables available at the census tract level can be aggregated to the county level; however, those at the county level cannot be aggregated to the census tract level. Researchers should advocate for more secondary data available at all levels (state, county, and census tract), making it more feasible to conduct quality research using secondary data. Further studies are needed to confirm these results because there is still a lack of studies addressing structural and intermediary SDOHs and maternal and infant mortality. Additionally, qualitative research designs would add a richer understanding of these complex associations through analysis of the experiences of pregnant and postpartum women [55].

Our study is not without limitations. These findings do not support causation; instead, we can only infer associations between the variables in the models. Based on the literature, structural and intermediary SDOH variables related to maternal and infant mortality and the most complete measures were included in the mediation model to predict maternal and infant mortality. The SDOH variables that performed best according to fit statistics were included in the models. This study uses county-level data, not individual-level data. For instance, although the outcome maternal mortality in Model 1 reflects events specific to women of reproductive age, many of the exposure variables (food insecurity, food assistance, walkability, mental distress, and some college attainment) are measured at the county population level and are not restricted to women of childbearing age. This limitation reflects the constraints of publicly available datasets, which typically report these indicators in aggregate. Consequently, these measures may only approximate the structural and contextual environments experienced by women of reproductive age rather than directly capturing their individual exposures. The use of area-based measures is common in population health research, but it introduces the potential for ecological fallacy, where associations observed at the county level may not fully translate to the individual level.

While this study highlights area-level associations between walkability, food insecurity, other SDOHs, and maternal and infant mortality in the US, the broader applicability of these findings should be interpreted cautiously. Maternal mortality in the United States is significantly higher than in other wealthy nations, reflecting unique structural, health system, and social inequities. Thus, our results may not fully translate to other high-income countries with different baseline maternal outcomes.

There are other potential confounders related to maternal and infant mortality, such as previous medical conditions (e.g., hypertension, DM), health-seeking behaviors, quality of prenatal care, substance use, or psychosocial problems that are not included in this study, and therefore the results should be interpreted with caution, as residual confounding may partly explain these findings. The CDC suppresses data for counties with fewer than ten maternal or infant deaths, which may potentially cause underestimation of maternal and infant mortality; therefore, we pooled multiple years of maternal and infant mortality data in our study to reduce suppression, which improved estimate stability but limits our ability to assess year-to-year changes.

Although reduced walkability had an observed association with maternal and infant mortality, these findings should be interpreted with caution. Walkability may function as a proxy for unmeasured individual- or community-level factors such as obesity, diet, or other lifestyle and health risk behaviors. Prior research has demonstrated strong associations between neighborhood walkability and obesity prevalence as well as physical activity levels. Because these variables were not directly measured in this study, it is not possible to disentangle the independent contribution of walkability from these related pathways. As such, the novelty of this finding may be limited, and future research should incorporate individual-level health behaviors to better determine the extent to which built environment features, such as walkability, independently influence maternal and infant health outcomes.

Walkability was assessed using data from a single year (2019), which was applied across the 2015–2020 analytic period. Although the built environment typically changes slowly and the index is designed to capture stable community features, this approach assumes minimal variability in walkability within counties. If substantial changes occurred during this period (e.g., rapid urban development or infrastructure investments), these would not be reflected in the measure.

While this study is subject to certain limitations, it provides important insights into the social and structural drivers of maternal and infant mortality. This study makes several contributions to the literature. Integrating multiple national datasets and pooling county-level data from 2015 to 2020 provides a more comprehensive analysis of maternal and infant mortality patterns than studies relying on a single data source. The use of structural equation modeling moves beyond descriptive analyses to test pathways linking structural social determinants of health, intermediary factors, and mortality outcomes. By examining outcomes at the county level, the study captures local variation that is often masked in state- or national-level analyses. Finally, the findings underscore specific structural and intermediary factors that may be targeted by policy and practice interventions to reduce persistent disparities in maternal and infant mortality across the United States.

Based on these findings, several actions are recommended to improve maternal and infant health. Policies should address food insecurity by expanding and improving programs like SNAP, WIC, and FMNP, ensuring access to nutritious foods, and reducing financial barriers through job training and education initiatives. Neighborhood walkability should be improved with more sidewalks, safe crossings, and bike lanes to reduce physical barriers to accessing food and health services. Policymakers should incorporate structural and intermediary social determinants of health, including income, education, and neighborhood environment, into strategies aimed at reducing maternal and infant mortality. Healthcare providers should screen women for social risks and connect them with resources such as food assistance programs, community services, and employment support. Finally, interventions should target multiple social determinants simultaneously, recognizing the interconnected nature of these factors and their combined observed impact on maternal and infant outcomes.

## 5. Conclusions

The maternal mortality rates are higher in the US compared to other high-income nations [4], and infant mortality rates in the US continue to rise [7]. Black women are particularly affected by maternal and infant mortalities [2,7]. These problems are serious public health issues requiring multifaceted approaches to improve over time. This study suggests food insecurity, food assistance, walkability, distance to obstetrics services, and married women are the structural and intermediary SDOHs that have significant observed associations with maternal mortality rates. Infant mortality rates have significant observed associations with food insecurity, food assistance, walkability, mental distress, post-secondary education, Black race, and annual median household income. This study extends prior research by integrating multiple national datasets to examine maternal and infant mortality within a structural equation modeling framework. By identifying the pathways through which structural and intermediary social determinants shape mortality outcomes, the findings highlight modifiable factors that can inform targeted policies and interventions to reduce disparities at the county level. These SDOHs need to be considered when revising and creating new policies to address these national health concerns to improve the quality and longevity of life for women and children in the US.

## Figures and Tables

**Table 1:** Exogenous and endogenous variables to explain maternal mortality.

Variable	Abbreviation	Data Sources
**Outcome variable**		
Total number of maternal deaths per 100,000 population (100,000 live births)	Maternal mortality	AHRQ SDOH 2015–2020–CDCW [27]
**Exogenous variables**		
Median age of female population	Median age female	AHRQ SDOH 2015–2020–ACS [27]
Percentage of population that is female	Female	AHRQ SDOH 2015–2020– ACS [27]
Percentage of population reporting Black or African American female	Black female	AHRQ SDOH 2015–2020– ACS [27]
Percentage of adults ages 25–44 with some post-secondary education	Some college	CHR 2015–2020—ACS [28]
Median household income, the income where half of households in a county earn more and half of households earn less	Household income	CHR 2015–2020—Small Area Income and Poverty Estimates [28]
Median distance in miles to the nearest obstetrics department, calculated using population weighted tract centroids in the county	Distance OB	AHRQ SDOH 2015–2020–POS [27]
Percentage of female population now married and spouse present (ages 15 and over)	Married	AHRQ SDOH 2015–2020–ACS [27]
**Endogenous variables**		
Percentage of population who lack adequate access to food	Food insecurity	CHR 2015–2020–Map the Meal Gap [28]
National Walkability Index	Walkability	EPA National Walkability Index 2019 [29]
Percentage of households that received food stamps/SNAP, past 12 months	Food assistance	AHRQ SDOH 2015–2020–ACS [27]
Frequent mental distress, percentage of adults reporting 14 or more days of poor mental health per month	Mental distress	CHR 2015–2020–BRFSS [28]

**Table 2:** Exogenous and endogenous variables to explain infant mortality.

Variable	Abbreviation	Data Sources
**Outcome variable**		
Number of all infant deaths (within 1 year), per 1000 live births	Infant mortality	CHR 2015–2020–NCHS Mortality Files [28]
**Exogenous variables**		
Median age of total population	Median age	AHRQ SDOH 2015–2020–ACS [27]
Percentage of population that is female	Female	AHRQ SDOH 2015–2020–ACS [27]
Percentage of population reporting Black or African American race alone	Race Black	AHRQ SDOH 2015–2020–ACS [27]
Percentage of adults ages 25–44 with some post-secondary education	Some college	CHR 2015–2020–ACS [28]
Median household income, the income where half of households in a county earn more and half of households earn less	Household income	CHR 2015–2020–Small Area Income and Poverty Estimates [28]
Median distance in miles to the nearest obstetrics department, calculated using population weighted tract centroids in the county	Distance OB	AHRQ SDOH 2015–2020–POS [27]
**Endogenous variables**		
Percentage of population who lack adequate access to food	Food insecurity	CHR 2015–2020–Map the Meal Gap [28]
National Walkability Index	Walkability	EPA National Walkability Index 2019 [29]
Percentage of households that received food stamps/SNAP, past 12 months	Food assistance	AHRQ SDOH 2015–2020–ACS [27]
Frequent mental distress, percentage of adults reporting 14 or more days of poor mental health per month	Mental distress	CHR 2015–2020–BRFSS [28]

**Table 3:** Descriptive statistics of exogenous and endogenous variables in the models.

Variables	# of Obs	Mean	Standard Deviation	Min	Max
Median age	18,846	41.22129	5.39347	21.4	68
Female	18,846	49.92361	2.402675	19.17	59.42
Race Black	18,846	9.035861	14.49487	0	87.79
Walkability	18,843	6.538606	2.081125	1.8	16.44444
Distance OB	18,846	11.02253	15.23759	0.13	468.84
Food insecurity	18,844	14.30758	4.110087	3	38
Some college	18,843	56.91756	11.69315	2.7	100
Food assistance	18,846	13.40371	6.567941	0	59.87
Household income	18,837	49,187.23	12,956.99	21,572	140,382
Mental distress	15,702	12.0719	2.051434	7	22
Married	18,846	48.73767	7.701039	14.5	100
Median age female	18,846	42.55538	5.504286	22.3	68.5
Black female	18,290	41.86479	19.06933	0	100
Infant mortality	7841	7.066497	2.334955	2	31
Maternal mortality	1136	0.6906074	0.3984674	0.13	2.77

# of Obs = number of observations.

**Table 4:** Direct effects of variables in SEM Model 1.

Variables	Estimate (Standard Error)	Critical Ratio	*p*-Value
**Exogenous**			
Median age female → Food insecurity	−0.028 (0.004)	−6.94	<0.001
Median age female → Food assistance	0.031 (0.006)	4.67	<0.001
Median age female → Walkability	−0.069 (0.002)	−28.04	<0.001
Median age female → Mental distress	−0.001 (0.002)	−0.61	0.540
Median age female → Maternal mortality	−0.002 (0.002)	−0.81	0.420
Female → Food insecurity	−0.041 (0.009)	−4.15	<0.001
Female → Food assistance	0.062 (0.015)	4.00	<0.001
Female → Mental distress	0.106 (0.005)	19.40	<0.001
Female → Walkability	0.087 (0.006)	14.42	<0.001
Black female → Food insecurity	0.014 (0.001)	12.25	<0.001
Black female → Food assistance	−0.002 (0.001)	−1.39	0.165
Black female → Walkability	−0.000 (0.000)	−0.03	0.977
Black female → Mental distress	0.002 (0.000)	3.12	0.002
Black female → Maternal mortality	0.001 (0.003)	0.59	0.555
Distance OB → Maternal mortality	0.045 (0.008)	5.42	<0.001
Married → Food insecurity	−0.188 (0.003)	−60.74	<0.001
Married → Food assistance	−0.384 (0.005)	−75.73	<0.001
Married → Mental distress	−0.067 (0.001)	−37.87	<0.001
Married → Maternal mortality	−0.014 (0.002)	−5.21	<0.001
Some college → Mental distress	−0.059 (0.001)	−46.25	<0.001
Some college → Food assistance	−0.124 (0.003)	−33.94	<0.001
Some college → Maternal mortality	0.000 (0.001)	0.58	0.564
Household income → Food insecurity	−0.000 (1.67 × 10^−6^)	−102.85	<0.001
Household income → Walkability	0.000 (1.05 × 10^−6^)	57.94	<0.001
Household income → Mental distress	−0.000 (1.15 × 10^−6^)	−42.42	<0.001
Household income → Food assistance	−0.000 (3.33 × 10^−6^)	−44.94	<0.001
Household income → Maternal mortality	8.31 × 10^−6^ (1.27 × 10^−6^)	0.66	0.511
**Endogenous**			
Food insecurity → Maternal mortality	0.028 (0.004)	7.04	<0.001
Food assistance → Maternal mortality	0.007 (0.003)	2.58	0.013
Mental distress → Maternal mortality	−0.011 (0.009)	−1.21	0.226
Walkability → Maternal mortality	−0.044 (0.004)	−9.20	<0.001

**Table 5:** Direct effects of variables in SEM Model 2.

Variables	Estimate (Standard Error)	Critical Ratio	*p*-Value
**Exogenous**			
Median age → Food insecurity	−0.085 (0.003)	−24.41	<0.001
Median age → Food assistance	−0.151 (0.006)	−22.29	<0.001
Median age → Mental distress	−0.031 (0.002)	−13.96	<0.001
Median age → Infant mortality	0.007 (0.004)	1.84	0.066
Median age → Walkability	−0.078 (0.002)	−30.54	<0.001
Female → Food insecurity	0.047 (0.008)	5.93	<0.001
Female → Walkability	0.102 (0.005)	18.14	<0.001
Female → Food assistance	0.220 (0.015)	14.30	<0.001
Female → Mental distress	0.143 (0.005)	27.69	<0.001
Race Black → Food insecurity	0.124 (0.001)	91.23	<0.001
Race Black → Walkability	−0.001 (0.000)	−1.98	0.048
Race Black → Food assistance	0.119 (0.002)	45.64	<0.001
Race Black → Mental distress	0.021 (0.000)	24.88	<0.001
Race Black → Infant mortality	0.047 (0.001)	25.99	<0.001
Some college → Food insecurity	−0.004 (0.002)	−2.18	0.029
Some college → Mental distress	−0.061 (0.001)	−46.27	<0.001
Some college → Food assistance	−0.130 (0.003)	−32.82	<0.001
Some college → Infant mortality	−0.013 (0.002)	−4.73	<0.001
Household income → Food insecurity	−0.000 (1.83 × 10^−6^)	−92.45	<0.001
Household income → Mental distress	−0.000 (1.16 × 10^−6^)	−46.24	<0.001
Household income → Food assistance	−0.000 (3.52 × 10^−6^)	−51.64	<0.001
Household income → Walkability	0.000 (1.08 × 10^−6^)	56.64	<0.001
Household income → Infant mortality	−0.000 (2.69 × 10^−6^)	−12.95	<0.001
Distance OB → Infant mortality	0.004 (0.002)	1.78	0.075
**Endogenous**			
Food insecurity → Infant mortality	0.065 (0.008)	7.36	<0.001
Walkability → Infant mortality	−0.120 (0.010)	−11.27	<0.001
Food assistance → Infant mortality	0.020 (0.004)	4.30	<0.001
Mental distress → Infant mortality	0.044 (0.017)	2.54	0.011

**Table 6:** Direct, indirect, and total effects of variables on maternal mortality.

Variable	Direct Effect	Indirect Effect	Total Effect
Median age female	−0.002416	0.0025529 ***	0.0001369
Female	0	−0.0058765 ***	−0.0058765 ***
Black female	0.0018544	0.0003695 ***	0.0022239
Walkability	−0.0446151 ***	0	−0.0446151 ***
Distance OB	0.0458938 ***	0	0.0458938 ***
Married	−0.0143204 ***	−0.007492 ***	−0.0218124 ***
Food insecurity	0.0283536 ***	0	0.0283536 ***
Some college	0.0008672	0.0002545	0.0006128
Food assistance	0.0076168 *	0	0.0076168 *
Household income	8.31 × 10^−6^	−8.16 × 10^−6^ ***	−7.33 × 10^−6^ ***
Mental distress	−0.0116919	0	−0.0116919

* *p* < 0.05, *** *p* < 0.001.

**Table 7:** Direct, indirect, and total effects of variables on infant mortality.

Variable	Direct Effect	Indirect Effect	Total Effect
Median age	0.0079941	−0.0006226	0.0073715
Female	0	0.0015134	0.0015134
Race Black	0.047153 ***	0.0117488 ***	0.0589018 ***
Walkability	−0.1207111 ***	0	−0.1200711 ***
Distance OB	0.0046756	0	0.0046756
Food insecurity	0.0654288 ***	0	0.0654288 ***
Some college	−0.0130635 ***	−0.0056365 ***	−0.0187 ***
Food assistance	0.0202814 ***	0	0.0202814 ***
Household income	−0.0000348 ***	−0.0000245 ***	−0.0000593 ***
Mental distress	0.0441203 **	0	0.0441203 **

** *p* < 0.01, *** *p* < 0.001.

## Data Availability

Data used in this study is publicly available through the EPA’s National Walkability Index: https://www.epa.gov/smartgrowth/smart-location-mapping (accessed on 7 August 2024), AHRQ’s SDOH database: https://www.ahrq.gov/sdoh/data-analytics/sdoh-data.html (accessed on 7 July 2024), and CHR: https://www.countyhealthrankings.org/health-data/methodology-and-sources/data-documentation (accessed on 7 July 2024).
